# Therapeutic Potential of Autologous Stem Cell Transplantation for Cerebral Palsy

**DOI:** 10.1155/2012/825289

**Published:** 2012-10-04

**Authors:** Chaitanya Purandare, D. G. Shitole, Vaijayantee Belle, Aarti Kedari, Neeta Bora, Meghnad Joshi

**Affiliations:** ^1^StemOne Biologicals Pvt. Ltd., Gat No. 801, Urwade, Gadewadi, Tal-Mulshi, Pune 412108, India; ^2^Shitole Hospital, Rajarampuri 1st Lane, Near Janata Bazar, Kolhapur 416008, India

## Abstract

*Background*. Cerebral palsy (CP) is a severe disabling disease with worldwide incidence being 2 to 3 per 1000 live births. CP was considered as a noncurable, nonreparative disorder, but stem cell therapy offers a potential treatment for CP. *Objective*. The present study evaluates the safety and efficacy of autologous bone-marrow-derived mononuclear cell (BMMNCs) transplantation in CP patient. *Material and Methods*. In the present study, five infusions of autologous stem cells were injected intrathecally. Changes in neurological deficits and improvements in function were assessed using Gross Motor Function Classification System (GMFCS-E&R) scale. *Results*. Significant motor, sensory, cognitive, and speech improvements were observed. Bowel and bladder control has been achieved. On the GMFCS-E&R level, the patient was promoted from grade III to I. *Conclusion*. In this study, we report that intrathecal infusion of autologous BMMNCs seems to be feasible, effective, and safe with encouraging functional outcome improvements in CP patient.

## 1. Introduction

CP is the commonest cause of severe neurological disability in children. The general prevalence is 2-3 per 1000 live births and has slightly increased in recent years. This is due to the decreased mortality of low-birth-weight infants together with an increased rate of cerebral palsy in the survivors [1]. CP describes a group of permanent disorders of the development of movement and posture, causing activity limitations. The motor disorders of cerebral palsy are often accompanied by disturbances of sensation, perception, cognition, communication, and behavior, by epilepsy, and by secondary musculoskeletal problems [2].

Treatment programs for CP encompass physical and behavioral therapy, pharmacologic and surgical treatments, mechanical aids, and management of associated medical conditions. While many of these treatments are helpful, none facilitate in reparative recovery of damaged brain. Recent advances in stem cell therapy provide the hope of developing more effective interventions in treating CP. Research has shown that bone-marrow-derived cells could develop into neural tissue [3, 4].Woodburyetal.claimed that adult rat and human bone marrow stromal cell differentiate into neurons [5]. Stem cell transplantation has been reported to be effective in animal models as well as in patients with other degenerative neurological disorders such as stroke and demyelination [6–8]. 

The present case study reports the intrathecal infusion of autologous BM-MNCs in a six-year-old CP patient. Thus, we hypothesize that using autologous bone-marrow-derived stem cells will provoke multiple regenerative responses and treat the underlying disease condition. 

## 2. Material and Methods

### 2.1. Patient Enrollment

A six-year-old girl diagnosed with CP was enrolled for the study. The patient was tested for various infectious disease markers. The study was initiated in May 2009 and the followup was taken for 24 months. A written informed consent was obtained from the patient's parents before the therapy. Permission for the present study was granted from the Institutional and Hospital ethics committee. Rehabilitation intervention like physiotherapy was continued after the stem cell therapy, which included self-range of motion, strengthening exercises, and balance exercises.

### 2.2. Procurement of Autologous Bone Marrow Cells

Bone marrow of the patient was extracted under general anesthesia (GA) from the posterior superior iliac crest by multiple aspirations in the operation theatre under aseptic conditions. 120 mL of bone marrow was collected in Bone Marrow Collection Bag containing sufficient volume of anticoagulant to avoid coagulation of the sample. 

### 2.3. Stem Cell Isolation

**Figure 1:**
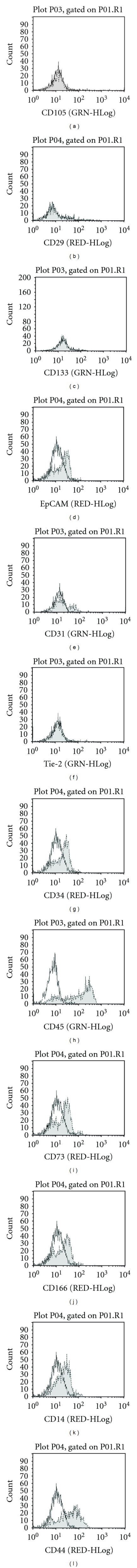
FACS characterization of transplanted cells. (a): (B:0%, T:100%), (b): HU(B:65, T:124), (c1): (B:0%, T:100%), (c2): (B: 85, T:165).

Microbiology testing of autologous bone marrow sample was done by an internationally accredited third party laboratory and was found to be negative. The sample was then sent to the laboratory for processing during which the BM-MNCs were separated using density gradient centrifugation method and characterized using BD FACS caliber flow cytometer (Figure 1 and Table 2). 

### 2.4. Mode of Infusion

The separated mononuclear cells were infused intrathecally (L_4_-L_5_) in aseptic conditions under short general anesthesia (GA). The transfusion lasted for 30 minutes and the child was discharged after 7-hour observation. No serious adverse effects after intrathecal infusions were observed (Table 1). 

### 2.5. Posttherapy Assessment

Posttherapy followup was done using the GMFCS-E&R scale on 1st, 3rd, 6th, and 12th month, and up to 2 years. Changes in neurological deficits and improvements in function were compared between pre- and post-therapy assessments using Gross Motor Function Classification System (GMFCS) scale between 6th and 12th birthdays.

## 3. Results 

### 3.1. Pretherapy Observations 

#### 3.1.1. History of Patient

An Indian first born female of G_1_-P_1_-L_1_-D_0_ was delivered via an uneventful caesarean section at 36 weeks with birth weight of 3.15 Kg. Her genetic studies showed 46 XX normal patterns. Neurosonography reports at second week showed tiny choroid plexus cyst. Magnetic resonance imaging (MRI) brain was performed which showed clinical impression of large head circumference, persistent cavum septum pellucidum, and no focal lesions within brain parenchyma. She had delayed milestones when examined at the age of 11 months and was diagnosed with cerebral palsy. In addition to hydrocephalus, tonsillar herniation was also seen, probably due to trans foraminal herniation secondary to increased intracranial pressure. At the age of 3 years, she showed the complain of convulsion, first in right upper limb and lower limb and later generalized. Continuous tonic/clonic with uprolling of eyeballs and twitching of angle of mouth episode lasted for 45 minutes. Electroencephalogram (EEG) showed bilateral epileptic form activity. Central nervous system (CNS) examination showed hypotonic muscle tone in left upper and lower limb.

#### 3.1.2. General Examination

General examination showed that the patient used to walk with support characterized by valgus deformity and was unable to hold things in hand. No bowel/bladder control was present. She was not able to recognize relatives. Speech was deformed; she suffered from word finding difficulty, and difficulty in conversation. She had good gross motor movements and coarse action. However, her fine movements were absent. Assessment on GMFCS-E&R scale showed grade III right and left UL and LL: power. Muscle tone was normal. The electroencephalogram (EEG) recording showed evidence of profuse multifocal epileptiform activity. 

#### 3.1.3. PET CT Scan Imaging

**Figure 2:**
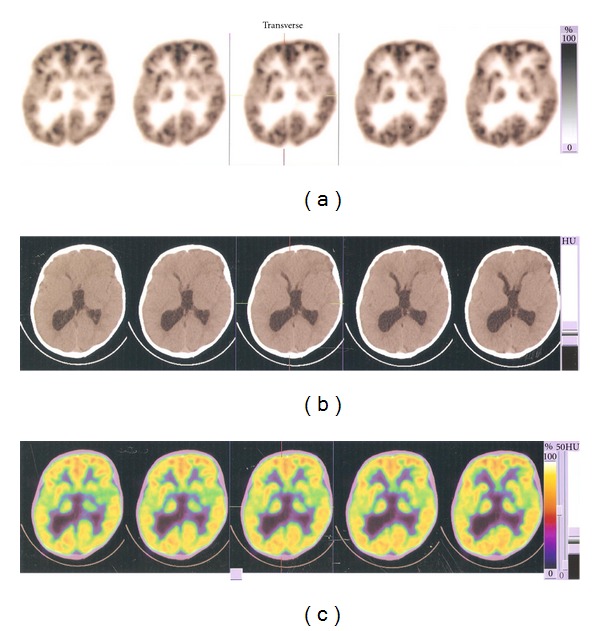
Pretherapy PET and CT scan findings: Third ventricle more dilated. Impaired motor, language social, and memory functions indicated by presence of more blue-purple area in the frontal lobe. (a): (B:0%, T:100%), (b): HU(B:65, T:124), (c1): (B:0%, T:100%), (c2): (B: 85, T:165).

Before the therapy, PET scan of the brain was done using Biograph Duo system. Both lateral and third ventricles appeared as mildly dilated and normal IV ventricles. Possibly, it represented partial aqueductal stenosis. Cerebellar tonsillar herniation was also seen. These features are seen in Arnold Chiari type I malformation.Tiny subcentimeter focal area of signal abnormality in right frontal parasagittal region is suggestive of chronic ischemic lesion (Figure 2).

### 3.2. Posttherapy Observations

#### 3.2.1. General Examination

**Motor Movements**

Within 6 months after interventions, she was able to walk without support but with slight valgus deformity. Presently, she is showing continuous motor improvements: can hold things in hand that is gripping is present, can hold crayons/pencils, and attempts to draw horizontal and vertical lines, and also draws figures. Thus, it can be inferred that there was significant improvement in fine motor movements. Initially, she had to be fed. Now, she is able to take the foodstuff up to her mouth on her own. On the GMFCS-E&R level, she can be promoted from grade III to grade I (Figure 4).

**Sensory Improvements**

The positive dynamics seen after the treatment was presence of eye contact. Fine hearing developed.

**Cognitive Improvements**

Her social know-how has improved significantly: recognizes relatives and remembers their names. She has developed problem and puzzle solving skills: can fix or indicate where the pieces will go in a puzzle and has good attention span when given a task. She has been promoted to higher grade in school.

**Speech Improvement**

Speech is improved. There is clarity in speech. She can speak complete sentences.

#### 3.2.2. PET Scan Imaging

**Figure 3:**
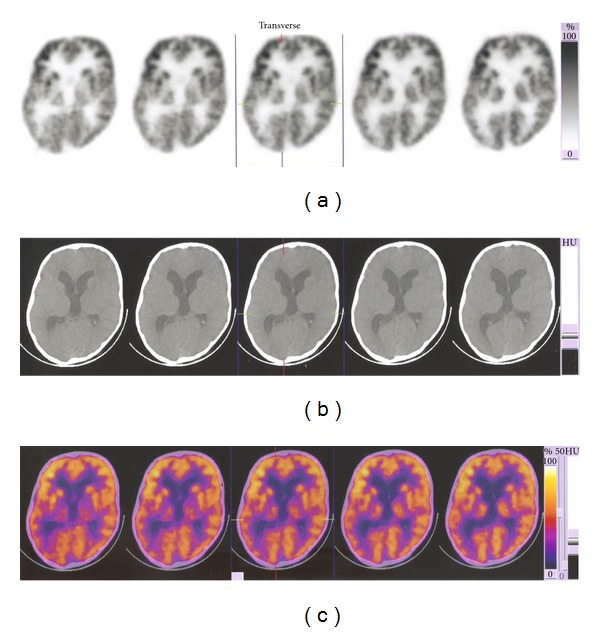
Posttherapy PET and CT scan findings: A slight decrease in the third ventricle dilation and increase in the yellow-orange area in the frontal lobe.

**Figure 4:**
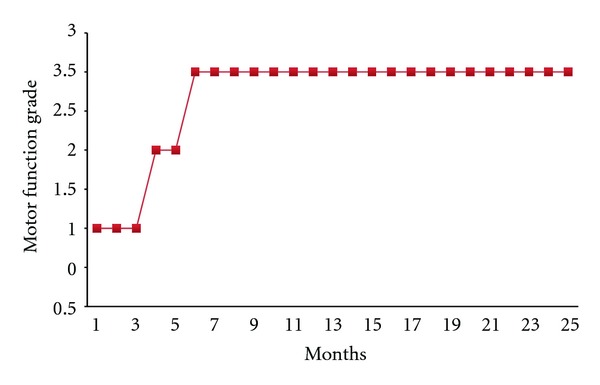
Graph showing gradewise posttherapy improvements in the patients.

A slight decrease in the third ventricle dilation is seen as compared to previous PET scan. Increase in the yellow-orange area in the frontal lobe compared to previous image was seen which attested to improved frontal lobe functioning (Figure 3).

According to EEG data, the child suffered from epilepsy and hence initially was given antiepileptics-Valparin syrup 7.5 mL twice a day and 2.5 mg frisium tablet daily. After 8 months of therapy, frisium dosage was reduced to 2.5 mg for alternate day. After 2 years of therapy, the frisium dosage was stopped completely. No epileptic episodes were observed.

## 4. Discussion

Satisfactory outcomes have not been achieved to date in treating CP by traditional therapies. CP represents a complex disorder and therefore series of interventions of effective therapeutics strategies are needed. Extensive research carried out in stem cell therapeutics has offered hope for conditions such as CP. By conducting this study, we provide the evidence of feasibility and efficacy of BMMNCs transplantation in CP patients. The results clearly show the improved neurological functions in the patient without any significant adverse effect. The transplanted cells can be used to replace damaged neurons, glia, and vasculature. The main findings of the study are the significant motor, cognitive, and sensory improvements in the patient.

Efficient delivery of stem cells at the site of injury plays a crucial role during cellular response. Basic animal and clinical experiment advocate, use of intrathecal route or lumbar puncture for stem cell delivery [8, 9]. Importantly, transplantation of cells is safe, less invasive, and no surgery part is involved. Transplanting cells into the subarachnoid space of the spinal cord will transport the cells through *cerebrospinal fluid* (CSF) and allow efficient delivery of cells at the site of injury [10, 11]. Another advantage of lumbar puncture is that cells can be transplanted multiple times to achieve significant number of cell replacement.

In the present clinical report, we transplanted first dose of BMMNCs (129.6 × 10^6^) by lumbar puncture immediately after enrichment and two doses at interval of seven days, fourth infusion after six months, and fifth infusion after one year. Repetitive lumbar puncture infusions in CSF increase the possibility of large number BMMNCs “homing” to the site of injury. The proposed mechanism may be that, first infusion likely to be exhausted to neutralize the toxic product or inflammatory reaction cascade. Transplanted cells also produce trophic factors and extracellular matrix, which will provide a permissive environment (neuroprotection) for next infusion of cells. Delivery of second BMMNCs through LP results in efficient “homing” to injury site. Furthermore, delivery of two more infusions of BMMNCs at seven-day interval increased engraftment efficiency that could make up for cell loss. Thus, probable success of functional outcome of BMMNCs transplantation can be the combination of these processes: (i) synthesis of neurotrophic/growth factors to support cell function and to prevent ongoing inflammatory cascade, (ii) prevention of cell death in neuronal population, (iii) establishment of vasculature, (iv) cell differentiation and integration, and (v) establishment of neuronal circuits and synaptic connectivity. Recently, Chopp and coworkers reported that rodent bone marrow cells grafted into the ischemic rat brain resulted in functional improvement [12, 13]. Another study done on lethally irradiated mice shows that stem cells originating from the bone marrow have the capacity to migrate across the blood brain barrier and to trans differentiate into microglia [13]. Previous *in vivo *studies have examined the differentiation of murine MSC. Transplantation of murine MSCs gave rise to neurons and astrocytes [4, 13]. Transplanted adult human bone marrow cells have been shown to enter the brain and generate neurons and this phenomenon could be exploited to prevent the development or progression of neurodegenerative diseases or to repair tissue damaged by infarction or trauma [14]. 

In this study, we have observed periventricular leukomalacia, inadequate and/or delayed myelination, shrinked white matter, and encephalomalacia in the CP child. CPs have abnormal neuroradiological findings, with white matter damage, the most common abnormality. Combined gray and white matter abnormalities are more common among children with hemiplegia; isolated white matter abnormalities, however, are always found with bilateral spasticity or athetosis, and with ataxia. Isolated gray matter damage, as reported, is the least common finding [15]. After transplantation of cells slight reduction has been observed in ventricle dilatation compared to the previous PET scan. Increase in yellow-orange area in frontal lobe region of the recent PET scan indicates that there is frontal lobe functioning improvement, which is consistent with our observation where fine motor movement and cognitive improvement has been noted. 

## 5. Conclusion

Our knowledge of the underlying mechanism of stem cells in the improvement of cerebral palsy deficits is in its infancy. Still the present study successfully provides the evidence of feasibility and efficacy of intrathecal stem cell transplantation. As autologous BMMNCs were used, there were no adverse effects, immunological reactions, and ethical issues. The definite improvements seen in the patient show great promise for cell transplantation as a therapy for cerebral palsy.

## Figures and Tables

**Table 1:** Infusion details.

Infusion number	Infusion	Cell count	Cell viability
1st	Day 1	129.6 × 10^6^	95.1%
2nd	Day 7	132.8 × 10^6^	90.39%
3rd	Day 14	90.6 × 10^6^	93.53%
4th	Month 6	121.5 × 10^6^	91.16%
5th	Month 12	86.9 × 10^6^	89.03%

**Table 2:** FACS analysis for surface markers expressed on BM-MNCs.

Infusion number	Mesenchymal cells	Epithelial cell	Endothelial cells	Monocytes	Lymphocytes
1	+	++	+	++++	+
2	+	+	+	++++	+
3	−	+	+	++++	+
4	−	+	+	++++	+
5	−	+	+	++++	+

(++++): highest percentage; (+): small percentage; (+): very small percentage; (−): cells absent.
